# 4,7-Diphenyl-2,9-bis­(trichloro­meth­yl)-1,10-phenanthroline

**DOI:** 10.1107/S1600536809051071

**Published:** 2009-12-04

**Authors:** Min-Hao Xie, Ya-Ling Liu, Pei Zou, Yong-Jun He, Biao Huang

**Affiliations:** a Jiangsu Institute of Nuclear Medicine, Wuxi 214063, People’s Republic of China

## Abstract

In the title compound, C_26_H_14_Cl_6_N_2_, the phenanthroline ring system is essentially planar, with an r.m.s. deviation of 0.048 (6) Å, and makes dihedral angles of 64.8 (14) and 66.6 (6)° with the two terminal phenyl rings. One of the trichloro­methyl groups is disordered over two positions, with occupancies of 0.42 (2) and 0.58 (2).

## Related literature

For 4,7-bis­(chloro­sulfophen­yl)-1,10-phenanthroline-2,9-dicarboxylic acid, see: Evangelista *et al.* (1988 ▶); Papanastasiou-Diamandi *et al.* (1989 ▶); Scorilas & Diamandis (2000 ▶). For a related structure, see: Wang *et al.* (2007 ▶).
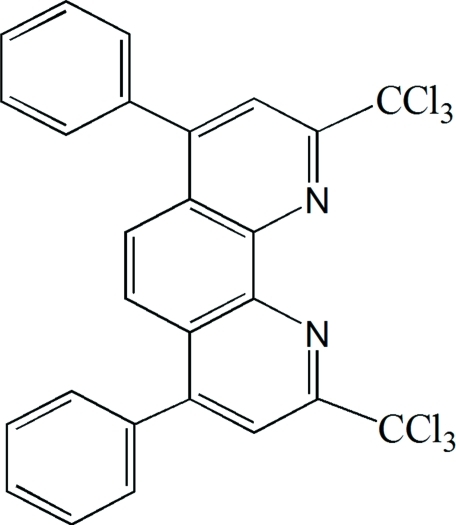


## Experimental

### 

#### Crystal data

C_26_H_14_Cl_6_N_2_
*M* *_r_* = 567.09
Monoclinic, 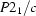

*a* = 11.253 (2) Å
*b* = 19.789 (4) Å
*c* = 11.299 (2) Å
β = 106.544 (3)°
*V* = 2411.9 (8) Å^3^
*Z* = 4
Mo *K*α radiation
μ = 0.73 mm^−1^
*T* = 133 K
0.30 × 0.27 × 0.20 mm

#### Data collection

Rigaku SPIDER diffractometer
Absorption correction: multi-scan (*ABSCOR*, Higashi, 1995 ▶) *T*_min_ = 0.810, *T*_max_ = 0.867
19334 measured reflections
5453 independent reflections
4573 reflections with *I* > 2σ(*I*)
*R*_int_ = 0.031

#### Refinement

*R*[*F* ^2^ > 2σ(*F* ^2^)] = 0.033
*wR*(*F* ^2^) = 0.080
*S* = 1.00
5453 reflections
335 parameters
6 restraints
H-atom parameters constrained
Δρ_max_ = 0.35 e Å^−3^
Δρ_min_ = −0.31 e Å^−3^

### 

Data collection: *RAPID-AUTO* (Rigaku 2004 ▶); cell refinement: *RAPID-AUTO*; data reduction: *RAPID-AUTO*; program(s) used to solve structure: *SHELXS97* (Sheldrick, 2008 ▶); program(s) used to refine structure: *SHELXL97* (Sheldrick, 2008 ▶); molecular graphics: *SHELXTL* (Sheldrick, 2008 ▶); software used to prepare material for publication: *SHELXL97*.

## Supplementary Material

Crystal structure: contains datablocks I, global. DOI: 10.1107/S1600536809051071/is2495sup1.cif

Structure factors: contains datablocks I. DOI: 10.1107/S1600536809051071/is2495Isup2.hkl

Additional supplementary materials:  crystallographic information; 3D view; checkCIF report

## Figures and Tables

**Fig. 1.**
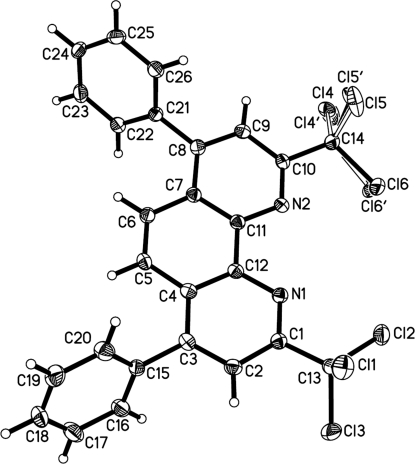
A view of the title compound with the atom-numbering scheme. Displacement ellipsoids were drawn at the 50% probability level and H atoms are represented as spheres of arbitrary radius.

## supplementary crystallographic information

### Comment

The molecule of the title complex (DDTP), (Fig.1), is an important intermediate for the synthesis of 4,7-bis(chlorosulfophenyl)-1,10- phenanthroline-2,9-dicarboxylic acid (BCPDA), a chelator that forms stable and highly fluorescent complexes with Eu^3+^ (Evangelista *et al.*, 1988). BCPDA can be covalently incorporated into proteins under relatively mild conditions (Papanastasiou-Diamandi *et al.*, 1989). and when complexes with Eu^3+^ forms a fluorescent product that has a lifetime in the range of 0.4 to 0.7 ms, it is useful for time-resolved fluorescence immunoassay applications (Scorilas & Diamandis, 2000). However, the crystal structure of DDTP has not been reported until now and therefore, we have determined its structure. In the crystal structure of the title compound, all bond lengths and angles are in good agreement with those observed in related compounds (Wang *et al.*, 2007). The phenanthroline ring is planar to within 0.048 (6) Å. The dihedral angles between the terminal phenyl rings and the phenanthroline unit are 64.8 (14) and 66.6 (6)°.

### Experimental

4,7-Diphenyl-2,9-dimethyl-1,10-phenanthroline (0.5 mmol, 180.2 mg), *N*-chlorosuccinimide (3.3 mmol, 440.6 mg) and benzoyl peroxide (0.5 mg) were dissolved in carbon tetrachlorid (6 ml). The reaction mixture was refluxed for 6 h. After cooling to room temperature, the reaction mixture was filtered. The filtrate was concentrated *in vacuo* and the residue was dissolved in chlorobenzene (3 mL). The solution was evaporated in air affording colourless block-shaped crystals suitable for X-ray analysis (yield: 80.1%).

### Refinement

H atoms were placed in calculated positions (C—H = 0.95 Å) and were refined using a riding model, with *U*_iso_(H) = 1.2*U*_eq_(C). The bond lengths of C14—Cl4, C14—Cl5, C14—Cl6, C14—Cl4', C14—Cl5' and C14—Cl6' were restrained to 1.777 (8) Å.

### Crystal data

**Table:**

C_26_H_14_Cl_6_N_2_	*F*(000) = 1144
*M**_r_* = 567.09	*D*_x_ = 1.562 Mg m^−^^3^
Monoclinic, *P*2_1_/*c*	Mo *K*α radiation, λ = 0.71073 Å
Hall symbol: -P 2ybc	Cell parameters from 7807 reflections
*a* = 11.253 (2) Å	θ = 3.0–27.5°
*b* = 19.789 (4) Å	µ = 0.73 mm^−^^1^
*c* = 11.299 (2) Å	*T* = 133 K
β = 106.544 (3)°	Block, colourless
*V* = 2411.9 (8) Å^3^	0.30 × 0.27 × 0.20 mm
*Z* = 4	

### Data collection

**Table:**

Rigaku SPIDER diffractometer	5453 independent reflections
Radiation source: Rotating Anode	4573 reflections with *I* > 2σ(*I*)
graphite	*R*_int_ = 0.031
ω scans	θ_max_ = 27.5°, θ_min_ = 3.1°
Absorption correction: multi-scan (*ABSCOR*, Higashi, 1995)	*h* = −14→14
*T*_min_ = 0.810, *T*_max_ = 0.867	*k* = −25→25
19334 measured reflections	*l* = −11→14

### Refinement

**Table:**

Refinement on *F*^2^	Primary atom site location: structure-invariant direct methods
Least-squares matrix: full	Secondary atom site location: difference Fourier map
*R*[*F*^2^ > 2σ(*F*^2^)] = 0.033	Hydrogen site location: inferred from neighbouring sites
*wR*(*F*^2^) = 0.080	H-atom parameters constrained
*S* = 1.00	*w* = 1/[σ^2^(*F*_o_^2^) + (0.0386*P*)^2^ + 0.845*P*] where *P* = (*F*_o_^2^ + 2*F*_c_^2^)/3
5453 reflections	(Δ/σ)_max_ = 0.001
335 parameters	Δρ_max_ = 0.35 e Å^−^^3^
6 restraints	Δρ_min_ = −0.31 e Å^−^^3^

### Special details

**Table:**

Geometry. All e.s.d.'s (except the e.s.d. in the dihedral angle between two l.s. planes) are estimated using the full covariance matrix. The cell e.s.d.'s are taken into account individually in the estimation of e.s.d.'s in distances, angles and torsion angles; correlations between e.s.d.'s in cell parameters are only used when they are defined by crystal symmetry. An approximate (isotropic) treatment of cell e.s.d.'s is used for estimating e.s.d.'s involving l.s. planes.
Refinement. Refinement of *F*^2^ against ALL reflections. The weighted *R*-factor *wR* and goodness of fit *S* are based on *F*^2^, conventional *R*-factors *R* are based on *F*, with *F* set to zero for negative *F*^2^. The threshold expression of *F*^2^ > σ(*F*^2^) is used only for calculating *R*-factors(gt) *etc*. and is not relevant to the choice of reflections for refinement. *R*-factors based on *F*^2^ are statistically about twice as large as those based on *F*, and *R*- factors based on ALL data will be even larger.

### Fractional atomic coordinates and isotropic or equivalent isotropic displacement parameters (Å^2^)

**Table:**

	*x*	*y*	*z*	*U*_iso_*/*U*_eq_	Occ. (<1)
Cl1	0.77991 (4)	0.20665 (2)	0.26183 (5)	0.03026 (12)	
Cl2	0.87385 (4)	0.34261 (2)	0.28505 (4)	0.02826 (11)	
Cl3	0.70433 (4)	0.29867 (3)	0.05663 (4)	0.03175 (12)	
Cl4	0.8632 (9)	0.5763 (2)	0.7849 (9)	0.0382 (18)	0.42 (2)
Cl5	0.9336 (8)	0.4395 (4)	0.8454 (7)	0.0288 (11)	0.42 (2)
Cl6	0.9431 (4)	0.4847 (7)	0.6140 (4)	0.0361 (12)	0.42 (2)
Cl4'	0.8577 (6)	0.57471 (16)	0.7930 (5)	0.0220 (7)	0.58 (2)
Cl5'	0.9403 (6)	0.4374 (3)	0.8367 (6)	0.0398 (12)	0.58 (2)
Cl6'	0.9246 (6)	0.5098 (5)	0.6005 (4)	0.0377 (11)	0.58 (2)
N1	0.65388 (13)	0.35117 (7)	0.36420 (13)	0.0176 (3)	
N2	0.69870 (13)	0.43350 (7)	0.56595 (13)	0.0189 (3)	
C1	0.63101 (15)	0.31204 (8)	0.26646 (15)	0.0184 (3)	
C2	0.51453 (16)	0.28295 (9)	0.20857 (16)	0.0206 (4)	
H2	0.5027	0.2567	0.1357	0.025*	
C3	0.41832 (15)	0.29307 (8)	0.25905 (16)	0.0187 (3)	
C4	0.44090 (15)	0.33316 (8)	0.36810 (15)	0.0175 (3)	
C5	0.34884 (15)	0.34379 (8)	0.43131 (15)	0.0186 (3)	
H5	0.2690	0.3243	0.3993	0.022*	
C6	0.37361 (15)	0.38123 (9)	0.53598 (15)	0.0189 (3)	
H6	0.3113	0.3867	0.5770	0.023*	
C7	0.49177 (15)	0.41274 (8)	0.58584 (15)	0.0177 (3)	
C8	0.52306 (15)	0.44919 (9)	0.69909 (16)	0.0193 (3)	
C9	0.63987 (16)	0.47611 (10)	0.74061 (16)	0.0237 (4)	
H9	0.6642	0.5002	0.8164	0.028*	
C10	0.72327 (16)	0.46772 (9)	0.66991 (16)	0.0212 (4)	
C11	0.58475 (15)	0.40500 (8)	0.52455 (15)	0.0174 (3)	
C12	0.55943 (15)	0.36274 (8)	0.41452 (15)	0.0167 (3)	
C13	0.74181 (16)	0.29291 (9)	0.22063 (16)	0.0200 (4)	
C14	0.85598 (15)	0.49422 (8)	0.72133 (13)	0.0259 (4)	
C15	0.29361 (15)	0.26235 (8)	0.20141 (16)	0.0185 (3)	
C16	0.22094 (17)	0.28428 (9)	0.08735 (16)	0.0244 (4)	
H16	0.2510	0.3187	0.0447	0.029*	
C17	0.10426 (18)	0.25642 (11)	0.03459 (19)	0.0320 (5)	
H17	0.0548	0.2721	−0.0433	0.038*	
C18	0.06033 (18)	0.20584 (11)	0.09570 (19)	0.0324 (5)	
H18	−0.0195	0.1869	0.0602	0.039*	
C19	0.13328 (19)	0.18308 (10)	0.20874 (18)	0.0308 (4)	
H19	0.1039	0.1479	0.2504	0.037*	
C20	0.24910 (18)	0.21126 (9)	0.26165 (17)	0.0259 (4)	
H20	0.2983	0.1955	0.3396	0.031*	
C21	0.43708 (15)	0.45433 (9)	0.77745 (15)	0.0186 (3)	
C22	0.32389 (16)	0.48808 (9)	0.73897 (16)	0.0218 (4)	
H22	0.2988	0.5088	0.6599	0.026*	
C23	0.24798 (16)	0.49136 (10)	0.81640 (17)	0.0252 (4)	
H23	0.1713	0.5149	0.7906	0.030*	
C24	0.28335 (17)	0.46052 (10)	0.93113 (17)	0.0253 (4)	
H24	0.2296	0.4616	0.9825	0.030*	
C25	0.39631 (18)	0.42821 (10)	0.97102 (17)	0.0252 (4)	
H25	0.4212	0.4079	1.0505	0.030*	
C26	0.47359 (16)	0.42539 (9)	0.89462 (16)	0.0217 (4)	
H26	0.5518	0.4036	0.9225	0.026*	

### Atomic displacement parameters (Å^2^)

**Table:**

	*U*^11^	*U*^22^	*U*^33^	*U*^12^	*U*^13^	*U*^23^
Cl1	0.0312 (2)	0.0216 (2)	0.0385 (3)	0.00736 (18)	0.0109 (2)	0.00183 (19)
Cl2	0.0198 (2)	0.0314 (2)	0.0348 (3)	−0.00346 (18)	0.00969 (19)	−0.00712 (19)
Cl3	0.0298 (2)	0.0472 (3)	0.0188 (2)	0.0067 (2)	0.00781 (19)	0.0009 (2)
Cl4	0.027 (2)	0.030 (2)	0.051 (4)	−0.0075 (14)	0.000 (2)	0.0095 (16)
Cl5	0.020 (2)	0.0270 (18)	0.032 (2)	0.0059 (11)	−0.0049 (14)	−0.0020 (12)
Cl6	0.0212 (9)	0.063 (3)	0.0263 (9)	−0.0124 (12)	0.0111 (8)	−0.0113 (14)
Cl4'	0.0240 (12)	0.0194 (11)	0.0206 (12)	−0.0074 (9)	0.0027 (9)	−0.0040 (9)
Cl5'	0.0176 (11)	0.0264 (13)	0.071 (3)	−0.0007 (9)	0.0053 (12)	−0.0114 (12)
Cl6'	0.0262 (11)	0.065 (2)	0.0277 (8)	−0.0212 (14)	0.0167 (8)	−0.0187 (10)
N1	0.0180 (7)	0.0173 (7)	0.0173 (7)	0.0013 (5)	0.0048 (6)	−0.0003 (6)
N2	0.0165 (7)	0.0230 (7)	0.0166 (7)	−0.0031 (6)	0.0037 (6)	−0.0025 (6)
C1	0.0188 (8)	0.0182 (8)	0.0181 (9)	0.0007 (6)	0.0050 (7)	0.0005 (7)
C2	0.0217 (9)	0.0206 (8)	0.0187 (9)	0.0002 (7)	0.0043 (7)	−0.0026 (7)
C3	0.0179 (8)	0.0157 (8)	0.0205 (9)	−0.0004 (6)	0.0024 (7)	0.0008 (7)
C4	0.0190 (8)	0.0157 (8)	0.0167 (8)	0.0008 (6)	0.0034 (7)	0.0013 (6)
C5	0.0158 (8)	0.0186 (8)	0.0201 (9)	−0.0020 (6)	0.0029 (7)	0.0012 (7)
C6	0.0174 (8)	0.0209 (8)	0.0185 (9)	0.0005 (6)	0.0054 (7)	0.0018 (7)
C7	0.0171 (8)	0.0185 (8)	0.0171 (9)	0.0002 (6)	0.0040 (7)	0.0013 (6)
C8	0.0180 (8)	0.0204 (8)	0.0195 (9)	−0.0003 (7)	0.0052 (7)	0.0003 (7)
C9	0.0212 (9)	0.0317 (10)	0.0178 (9)	−0.0057 (7)	0.0051 (7)	−0.0079 (7)
C10	0.0160 (8)	0.0276 (9)	0.0193 (9)	−0.0058 (7)	0.0041 (7)	−0.0031 (7)
C11	0.0172 (8)	0.0181 (8)	0.0162 (8)	−0.0008 (6)	0.0037 (6)	0.0014 (6)
C12	0.0179 (8)	0.0159 (8)	0.0154 (8)	0.0007 (6)	0.0031 (6)	0.0025 (6)
C13	0.0199 (8)	0.0210 (8)	0.0177 (9)	0.0010 (7)	0.0032 (7)	−0.0014 (7)
C14	0.0199 (9)	0.0382 (11)	0.0210 (10)	−0.0082 (8)	0.0079 (7)	−0.0083 (8)
C15	0.0175 (8)	0.0175 (8)	0.0208 (9)	−0.0016 (6)	0.0059 (7)	−0.0049 (7)
C16	0.0245 (9)	0.0252 (9)	0.0218 (9)	−0.0037 (7)	0.0038 (7)	−0.0008 (7)
C17	0.0262 (10)	0.0381 (11)	0.0262 (11)	−0.0041 (8)	−0.0017 (8)	−0.0020 (8)
C18	0.0225 (10)	0.0388 (12)	0.0356 (12)	−0.0122 (8)	0.0076 (8)	−0.0113 (9)
C19	0.0346 (11)	0.0278 (10)	0.0339 (11)	−0.0129 (8)	0.0161 (9)	−0.0035 (8)
C20	0.0284 (10)	0.0243 (9)	0.0235 (10)	−0.0031 (7)	0.0048 (8)	0.0009 (7)
C21	0.0185 (8)	0.0207 (8)	0.0173 (9)	−0.0043 (6)	0.0063 (7)	−0.0039 (7)
C22	0.0191 (9)	0.0255 (9)	0.0198 (9)	−0.0026 (7)	0.0040 (7)	0.0006 (7)
C23	0.0168 (8)	0.0292 (10)	0.0297 (10)	−0.0009 (7)	0.0067 (7)	−0.0046 (8)
C24	0.0232 (9)	0.0341 (10)	0.0223 (10)	−0.0089 (8)	0.0123 (7)	−0.0079 (8)
C25	0.0302 (10)	0.0291 (10)	0.0161 (9)	−0.0082 (8)	0.0063 (7)	−0.0007 (7)
C26	0.0199 (8)	0.0230 (9)	0.0210 (9)	−0.0005 (7)	0.0040 (7)	0.0002 (7)

### Geometric parameters (Å, °)

**Table:**

Cl1—C13	1.7890 (18)	C8—C21	1.489 (2)
Cl2—C13	1.7575 (18)	C9—C10	1.405 (2)
Cl3—C13	1.7831 (18)	C9—H9	0.9500
Cl4—C14	1.769 (3)	C10—C14	1.533 (2)
Cl5—C14	1.791 (3)	C11—C12	1.458 (2)
Cl6—C14	1.773 (3)	C15—C16	1.385 (2)
Cl4'—C14	1.784 (2)	C15—C20	1.389 (2)
Cl5'—C14	1.777 (2)	C16—C17	1.392 (3)
Cl6'—C14	1.777 (2)	C16—H16	0.9500
N1—C1	1.313 (2)	C17—C18	1.385 (3)
N1—C12	1.360 (2)	C17—H17	0.9500
N2—C10	1.315 (2)	C18—C19	1.382 (3)
N2—C11	1.356 (2)	C18—H18	0.9500
C1—C2	1.410 (2)	C19—C20	1.387 (3)
C1—C13	1.528 (2)	C19—H19	0.9500
C2—C3	1.375 (2)	C20—H20	0.9500
C2—H2	0.9500	C21—C26	1.393 (2)
C3—C4	1.426 (2)	C21—C22	1.394 (2)
C3—C15	1.498 (2)	C22—C23	1.388 (2)
C4—C12	1.414 (2)	C22—H22	0.9500
C4—C5	1.431 (2)	C23—C24	1.385 (3)
C5—C6	1.356 (2)	C23—H23	0.9500
C5—H5	0.9500	C24—C25	1.379 (3)
C6—C7	1.431 (2)	C24—H24	0.9500
C6—H6	0.9500	C25—C26	1.390 (2)
C7—C11	1.418 (2)	C25—H25	0.9500
C7—C8	1.423 (2)	C26—H26	0.9500
C8—C9	1.371 (2)		
			
C1—N1—C12	117.49 (14)	Cl4—C14—Cl6	113.9 (4)
C10—N2—C11	117.37 (14)	C10—C14—Cl6'	110.97 (16)
N1—C1—C2	124.37 (15)	C10—C14—Cl5'	108.7 (2)
N1—C1—C13	116.60 (14)	Cl6'—C14—Cl5'	114.5 (4)
C2—C1—C13	118.83 (15)	C10—C14—Cl4'	111.2 (2)
C3—C2—C1	119.08 (16)	Cl6'—C14—Cl4'	104.1 (3)
C3—C2—H2	120.5	Cl5'—C14—Cl4'	107.3 (3)
C1—C2—H2	120.5	C10—C14—Cl5	107.2 (3)
C2—C3—C4	118.16 (15)	Cl4—C14—Cl5	106.4 (4)
C2—C3—C15	120.78 (15)	Cl6—C14—Cl5	103.3 (5)
C4—C3—C15	121.07 (15)	C16—C15—C20	118.96 (16)
C12—C4—C3	117.91 (15)	C16—C15—C3	120.62 (15)
C12—C4—C5	119.64 (15)	C20—C15—C3	120.42 (16)
C3—C4—C5	122.45 (15)	C15—C16—C17	120.63 (17)
C6—C5—C4	121.15 (15)	C15—C16—H16	119.7
C6—C5—H5	119.4	C17—C16—H16	119.7
C4—C5—H5	119.4	C18—C17—C16	120.00 (19)
C5—C6—C7	121.25 (16)	C18—C17—H17	120.0
C5—C6—H6	119.4	C16—C17—H17	120.0
C7—C6—H6	119.4	C19—C18—C17	119.60 (18)
C11—C7—C8	117.76 (15)	C19—C18—H18	120.2
C11—C7—C6	119.54 (15)	C17—C18—H18	120.2
C8—C7—C6	122.62 (15)	C18—C19—C20	120.34 (18)
C9—C8—C7	118.18 (15)	C18—C19—H19	119.8
C9—C8—C21	119.46 (15)	C20—C19—H19	119.8
C7—C8—C21	122.20 (15)	C19—C20—C15	120.47 (18)
C8—C9—C10	119.34 (16)	C19—C20—H20	119.8
C8—C9—H9	120.3	C15—C20—H20	119.8
C10—C9—H9	120.3	C26—C21—C22	119.32 (16)
N2—C10—C9	124.34 (16)	C26—C21—C8	118.11 (15)
N2—C10—C14	116.68 (14)	C22—C21—C8	122.55 (15)
C9—C10—C14	118.73 (14)	C23—C22—C21	119.86 (16)
N2—C11—C7	122.92 (15)	C23—C22—H22	120.1
N2—C11—C12	117.95 (14)	C21—C22—H22	120.1
C7—C11—C12	119.10 (15)	C24—C23—C22	120.34 (17)
N1—C12—C4	122.85 (15)	C24—C23—H23	119.8
N1—C12—C11	117.83 (14)	C22—C23—H23	119.8
C4—C12—C11	119.25 (15)	C25—C24—C23	120.19 (16)
C1—C13—Cl2	113.35 (12)	C25—C24—H24	119.9
C1—C13—Cl3	111.23 (12)	C23—C24—H24	119.9
Cl2—C13—Cl3	108.45 (9)	C24—C25—C26	119.82 (17)
C1—C13—Cl1	107.85 (12)	C24—C25—H25	120.1
Cl2—C13—Cl1	108.33 (9)	C26—C25—H25	120.1
Cl3—C13—Cl1	107.44 (9)	C25—C26—C21	120.42 (17)
C10—C14—Cl4	113.3 (4)	C25—C26—H26	119.8
C10—C14—Cl6	111.90 (18)	C21—C26—H26	119.8
			
C12—N1—C1—C2	1.9 (2)	C2—C1—C13—Cl2	170.74 (13)
C12—N1—C1—C13	−172.95 (14)	N1—C1—C13—Cl3	−136.63 (14)
N1—C1—C2—C3	−3.0 (3)	C2—C1—C13—Cl3	48.23 (19)
C13—C1—C2—C3	171.78 (15)	N1—C1—C13—Cl1	105.80 (15)
C1—C2—C3—C4	0.4 (2)	C2—C1—C13—Cl1	−69.34 (18)
C1—C2—C3—C15	−179.20 (15)	N2—C10—C14—Cl4	140.4 (4)
C2—C3—C4—C12	2.9 (2)	C9—C10—C14—Cl4	−45.1 (4)
C15—C3—C4—C12	−177.55 (15)	N2—C10—C14—Cl6	9.9 (5)
C2—C3—C4—C5	−176.69 (16)	C9—C10—C14—Cl6	−175.5 (5)
C15—C3—C4—C5	2.9 (2)	N2—C10—C14—Cl6'	28.8 (4)
C12—C4—C5—C6	−0.7 (2)	C9—C10—C14—Cl6'	−156.6 (4)
C3—C4—C5—C6	178.88 (16)	N2—C10—C14—Cl5'	−98.0 (3)
C4—C5—C6—C7	1.3 (3)	C9—C10—C14—Cl5'	76.6 (4)
C5—C6—C7—C11	0.3 (2)	N2—C10—C14—Cl4'	144.1 (3)
C5—C6—C7—C8	−176.41 (16)	C9—C10—C14—Cl4'	−41.3 (3)
C11—C7—C8—C9	1.7 (2)	N2—C10—C14—Cl5	−102.6 (4)
C6—C7—C8—C9	178.40 (17)	C9—C10—C14—Cl5	71.9 (4)
C11—C7—C8—C21	−173.61 (15)	C2—C3—C15—C16	−68.2 (2)
C6—C7—C8—C21	3.1 (3)	C4—C3—C15—C16	112.29 (19)
C7—C8—C9—C10	0.9 (3)	C2—C3—C15—C20	111.8 (2)
C21—C8—C9—C10	176.27 (17)	C4—C3—C15—C20	−67.8 (2)
C11—N2—C10—C9	0.5 (3)	C20—C15—C16—C17	1.0 (3)
C11—N2—C10—C14	174.73 (14)	C3—C15—C16—C17	−179.04 (17)
C8—C9—C10—N2	−2.1 (3)	C15—C16—C17—C18	−0.6 (3)
C8—C9—C10—C14	−176.22 (16)	C16—C17—C18—C19	−0.4 (3)
C10—N2—C11—C7	2.3 (2)	C17—C18—C19—C20	0.9 (3)
C10—N2—C11—C12	−175.49 (15)	C18—C19—C20—C15	−0.4 (3)
C8—C7—C11—N2	−3.4 (2)	C16—C15—C20—C19	−0.5 (3)
C6—C7—C11—N2	179.78 (15)	C3—C15—C20—C19	179.56 (17)
C8—C7—C11—C12	174.39 (15)	C9—C8—C21—C26	−59.3 (2)
C6—C7—C11—C12	−2.5 (2)	C7—C8—C21—C26	115.88 (19)
C1—N1—C12—C4	1.7 (2)	C9—C8—C21—C22	119.2 (2)
C1—N1—C12—C11	178.76 (15)	C7—C8—C21—C22	−65.6 (2)
C3—C4—C12—N1	−4.1 (2)	C26—C21—C22—C23	−1.3 (3)
C5—C4—C12—N1	175.48 (15)	C8—C21—C22—C23	−179.75 (16)
C3—C4—C12—C11	178.89 (15)	C21—C22—C23—C24	−0.8 (3)
C5—C4—C12—C11	−1.5 (2)	C22—C23—C24—C25	2.2 (3)
N2—C11—C12—N1	3.8 (2)	C23—C24—C25—C26	−1.4 (3)
C7—C11—C12—N1	−174.08 (15)	C24—C25—C26—C21	−0.7 (3)
N2—C11—C12—C4	−179.07 (15)	C22—C21—C26—C25	2.0 (3)
C7—C11—C12—C4	3.1 (2)	C8—C21—C26—C25	−179.40 (16)
N1—C1—C13—Cl2	−14.1 (2)		
